# No Expression Divergence despite Transcriptional Interference between Nested Protein-Coding Genes in Mammals

**DOI:** 10.3390/genes12091381

**Published:** 2021-09-01

**Authors:** Raquel Assis

**Affiliations:** Department of Electrical Engineering and Computer Science, Institute for Human Health and Disease Intervention, Florida Atlantic University, Boca Raton, FL 33431, USA; rassis@fau.edu

**Keywords:** nested genes, overlapping genes, transcriptional interference, gene expression

## Abstract

Nested protein-coding genes accumulated throughout metazoan evolution, with early analyses of human and *Drosophila* microarray data indicating that this phenomenon was simply due to the presence of large introns. However, a recent study employing RNA-seq data uncovered evidence of transcriptional interference driving rapid expression divergence between *Drosophila* nested genes, illustrating that accurate expression estimation of overlapping genes can enhance detection of their relationships. Hence, here I apply an analogous approach to strand-specific RNA-seq data from human and mouse to revisit the role of transcriptional interference in the evolution of mammalian nested genes. A genomic survey reveals that whereas mammalian nested genes indeed accrued over evolutionary time, they are retained at lower frequencies than in *Drosophila*. Though several properties of mammalian nested genes align with observations in *Drosophila* and with expectations under transcriptional interference, contrary to both, their expression divergence is not statistically different from that between unnested genes, and also does not increase after nesting. Together, these results support the hypothesis that lower selection efficiencies limit rates of gene expression evolution in mammals, leading to their reliance on immediate eradication of deleterious nested genes to avoid transcriptional interference.

## 1. Introduction

Surveys of eukaryotic genome architecture have uncovered high frequencies of nested protein-coding genes, in which one “internal” gene is located in an intron of a second “external” gene [1,2,3,4,5,6]. Internal genes are typically short and intronless, whereas external genes tend to be long and possess many large introns [2,3,4]. A comprehensive analysis across three metazoan lineages illustrated that internal genes often arise via gene duplication, and that nested genes are formed when the resulting young duplicate genes are inserted into introns of existing genes [3]. This study also revealed that nested genes accumulated over evolutionary time, as evidenced by the predominance of nesting relative to unnesting events in all three metazoan lineages [3].

The finding that frequencies of nested genes increased over evolutionary time [3] is surprising, as such structures are expected to be evolutionarily disfavored due to transcriptional interference between external and internal genes [7,8]. Indeed, most external and internal genes are transcribed from opposite strands [2,4,5,6]. Nevertheless, interrogations of early human [9] and *Drosophila melanogaster* [10] microarray data yielded positive correlations between expression profiles of nested genes [3,6]. Though smaller than positive correlations between expression profiles of adjacent genes, they were found to be no different than those between intra-chromosomal genes [3,6]. Thus, these results support the hypothesis that nested genes accumulated over time simply because of increased nesting opportunities provided by large metazoan introns [3].

Yet, the conclusions of these early studies [3,6] were clouded by their dependence on data from microarray experiments, which can yield inaccurate estimates of gene expression levels for overlapping genes. Moreover, the usage of correlation coefficients to assess expression divergence is biased when measurement error is large [11]. With these limitations in mind, a recent study used RNA-seq data [12,13] and Euclidian distance estimates of gene expression divergence [11] to reexamine the hypothesis that transcriptional interference impacts nested gene evolution in *Drosophila* [6]. This analysis uncovered widespread expression divergence between nested genes that was greater than that between either intra- or inter-chromosomal genes, providing strong support for transcriptional interference between nested genes in *Drosophila* [6]. Further, both expression and sequence divergence were found to rapidly increase after nesting, indicating that natural selection plays an important role in avoidance of transcriptional interference between *Drosophila* nested genes [6]. 

These findings in *Drosophila* prompt the question of whether transcriptional interference drives the evolution of nested genes in other taxonomic groups. Thus, here I address this question in mammals, which also accumulated nested genes over evolutionary time [3]. To do so, I take advantage of high-quality genome sequence and annotation data, along with strand-specific RNA-seq data from the same seven tissues [14,15], in human and mouse. Following the approach taken in *Drosophila* [6], I investigate mammalian nested gene prevalence and evolutionary dynamics, genomic and transcriptomic properties, and expression divergence. Joint consideration of these findings allows me to assess whether and how transcriptional interference influences the evolution of nested genes in the mammalian lineage. 

## 2. Materials and Methods

### 2.1. Identification of Nested and Unnested Gene Pairs

Genome annotation (gtf) files for human (*Homo sapiens*), mouse (*Mus musculus*), cow (*Bos taurus*), opossum (*Monodelphis domestica*), platypus (*Ornithorhynchus anatinus*), chicken (*Gallus gallus*), and zebrafish (*Danio rerio*) were retrieved from the Ensembl release 104 [16] FTP site at ftp.ensembl.org (accessed on 23 August 2021). There are 452 pairs of nested protein-coding genes annotated in human (4.4%), 484 in mouse (4.3%), 745 in cow (6.8%), 926 in opossum (8.7%), 521 in platypus (6.0%), 640 in chicken (7.6%), and 673 in zebrafish (5.3%). The number of human nested genes identified here is consistent with that obtained from an older genome assembly [3]. For this study, I focused on properties of nested genes in human (Table S1) and mouse (Table S2), which have high-quality genome assemblies and annotation data sets, similar proportions of annotated nested genes, and strand-specific RNA-seq data from the same seven tissues (see Section 2.3 below). For comparison, I also obtained all intra-chromosomal (8,033,791 in human and 11,247,158 in mouse) and inter-chromosomal (142,486,134 in human and 190,692,335 in mouse) protein-coding gene pairs from the 17,351 and 20,113 unnested and non-overlapping protein-coding genes annotated in human and mouse, respectively. The null model for all comparisons between nested and unnested genes in this study is that their properties are similar, as that is the expectation in the absence of transcriptional interference. 

### 2.2. Inference of Gene Nesting and Unnesting Events

I obtained 1:1 orthologs for all protein-coding genes in human (*Homo sapiens*), mouse (*Mus musculus*), cow (*Bos taurus*), opossum (*Monodelphis domestica*), platypus (*Ornithorhynchus anatinus*), chicken (*Gallus* gallus), and zebrafish (*Danio rerio*) from Ensembl release 104 [16] via the BioMart database [17]. Nesting events that occurred before the divergence of human and mouse lineages were inferred based on their presence in both of these species. In contrast, nesting events that occurred after the divergence of human and mouse lineages were inferred based on their presence in only one of these species and their absence in all outgroups. Though it is possible that genes underwent nesting and unnesting multiple times throughout evolution, the stringent requirement that nesting be absent in all outgroups enabled conservative identification of human- or mouse-specific nesting events. Moreover, to ensure that incomplete genome assembly or annotation errors did not bias the inference of such nesting events, I required that external and internal genes both have orthologs in human, mouse, and at least one outgroup. Thus, nesting events were not inferred when one or both genes are simply absent ancestrally. 

### 2.3. Gene Expression Analyses

Tables of normalized strand-specific RNA-seq abundances in transcripts per million (TPM) from brain, lung, liver, spleen, kidney, colon, and testis tissues in human (E-MTAB-4344) [15] and mouse (E-MTAB-2801) [14] were downloaded from Expression Atlas [18] at https://www.ebi.ac.uk/gxa/home/ (accessed on 23 August 2021). All data in Expression Atlas are obtained with the iRAP pipeline, averaged across technical replicates, and quantile normalized [19]. Though there are numerous gene expression data sets available for human and mouse, I chose these specifically because they contain seven of the same tissues and were obtained from strand-specific RNA-seq experiments, which enable more accurate expression quantification of overlapping genes [20]. To minimize noise, all genes with TPM ≥1 in at least one of the seven tissues were retained for expression analyses, yielding 269 human (Table S1) and 265 mouse (Table S2) nested genes for which both external and internal genes met this threshold. The requirement that both external and internal genes be expressed was used to ensure that findings from expression analyses involving one and both genes are comparable, and also that transcriptional interference between the genes is possible. I estimated the expression breadth of each gene by computing the tissue specificity index τ, which ranges from 0 (broadly expressed) to 1 (tissue specific [21]), and the expression divergence between each pair of genes by computing the Euclidian distance between their relative TPM across tissues, which enables inter-species comparisons [11,22]. 

### 2.4. Statistical Analyses

All statistical analyses were performed in the R software environment [23]. Two-tailed binomial tests implemented with the binom.test() function in the stats package [23] were used to compare numbers of nested genes on the same vs. opposite strands, numbers of tissue-specific external vs. internal genes, and numbers of tissue-specific genes expressed in each tissue for nested vs. unnested genes. In each comparison of nested genes on the same vs. opposite strands, *x* was set to the number of nested genes on opposite strands, *n* to the total number of nested genes, and *p* = 0.5 to represent the expected frequency of opposite-strand nestings if orientation is random. In each comparison of numbers of tissue-specific external vs. internal genes, x was set to the number of tissue-specific external genes, n to the total number of tissue-specific external and internal genes, and *p* = 0.5 to represent the expected frequency of tissue-specific external genes if tissue specificity is random. In each comparison of numbers of tissue-specific genes expressed in each tissue for nested vs. unnested genes, *x* was set to the number of tissue-specific nested genes in the tissue of interest, n to the total number of tissue-specific nested genes, and p to the proportion of tissue-specific unnested genes in the tissue of interest. For these analyses, *p*-values were Bonferroni-corrected for the seven comparisons performed. Two-tailed Fisher’s exact tests implemented with the fisher.test() function in the stats package [23] were used to compare numbers of nested genes on the same vs. opposite strands and numbers of tissue-specific external vs. internal genes between human and mouse. Two-tailed two-sample permutation tests implemented with the permTS() function in the perm package [24] were used for all pairwise comparisons between distributions. For comparisons involving intra- or inter-chromosomal gene pairs, the permControl() function was used to restrict the number of permutations to 1000. 

## 3. Results

### 3.1. Prevalence and Evolutionary Dynamics of Nested Protein-Coding Genes in Mammals

Across the seven vertebrate species surveyed, which included five mammals, 4.3–8.7% of protein-coding genes were found in nested structures (see Materials and Methods for details). Human and mouse genomes sit at the lower end of this range, with 4.4% and 4.3% of their genes nested, respectively. These proportions are roughly half of those observed across 12 *Drosophila* species [6], consistent with relative frequencies obtained in an earlier study of metazoan nested genes [3]. Thus, also taking into consideration that human and mouse have the highest quality and best annotated genomes among those of the species examined here, gene nesting appears to be much less common in mammals than in *Drosophila*. Because most nested genes arise from the insertion of young duplicate genes into the introns of existing genes [3], this difference may be attributed to either gene duplication or nesting. However, the higher gene duplication rates in mammals [25,26] and similar proportions of retained duplicate genes in mammalian and *Drosophila* genomes [27] are inconsistent with a difference due to either neutral or selective forces involved in gene duplication. A neutral scenario in which genomic composition impacts nesting probabilities is also unlikely, as intronic and intergenic regions display conserved 1:1 ratios across metazoans [28]. As a result, the lower frequency of nested genes in mammals may be best explained by stronger selection to eradicate such structures, which is likely the first mechanism of defense against transcriptional interference between external and internal genes. 

Analysis of the evolutionary dynamics of human and mouse nested protein-coding genes (see Materials and Methods for details) uncovered 73 (Table S3) nesting events that occurred before the divergence of the two mammalian lineages, and 56 nesting events that occurred after their divergence—34 in the human lineage (Table S4) and 22 in the mouse lineage (Table S5). In contrast, I only identified four cases of unnesting events (three in human and one in mouse), mirroring previous findings of more frequent nesting than unnesting in human and mouse [3]. Because this earlier study also revealed a similar trend in *Drosophila* and *Caenorhabditis* lineages [3], the current analysis supports the hypothesis that gene nestings accumulate and contribute to the increased organizational complexity of mammalian and other metazoan genomes over evolutionary time [3]. This phenomenon was previously explained by the presence of large metazoan introns [3], which take up as much genomic space as intergenic regions [28] and offer ample opportunities for gene nesting. However, another contributing factor may be rapid gene duplication, as this mutational process creates most internal genes [3]. Indeed, experimental studies have shown that gene duplication occurs faster than all other types of spontaneous mutation in several metazoan species [26,29,30,31,32]. Hence, if this pattern holds in mammals, then it is possible that large and abundant mammalian introns provide much-needed homes for floods of newly generated young duplicate genes. 

### 3.2. Genomic and Transcriptomic Properties of Nested Protein-Coding Genes in Mammals

Though large introns coupled with fast duplication rates may contribute to the rapid creation of mammalian nested protein-coding genes, it is curious how such structures persist in the presence of transcriptional interference between external and internal genes. As a first step in addressing this question, I examined relationships between external and internal genes. To facilitate direct 1:1 comparisons between external and internal genes, I restricted my analysis to the 296 human (Table S1) and 248 mouse (Table S2) simple nested protein-coding gene pairs, in which an external gene contains only one internal gene in its intron. Of these simple nested gene pairs, 220 in human (74.3%) and 193 in mouse (77.8%) contain external and internal genes on opposite strands (Table 1). Hence, there are similar opposite-strand biases in human and mouse, consistent with those observed in previous studies of human [2] and *Drosophila* [6] nested genes. These comparably strong biases point to a preference for opposite-strand nestings that crosses taxonomic boundaries, suggesting that purging of same-strand nestings by negative selection may serve as a global mechanism for reducing transcriptional interference between nested genes. 

**Figure 1:**
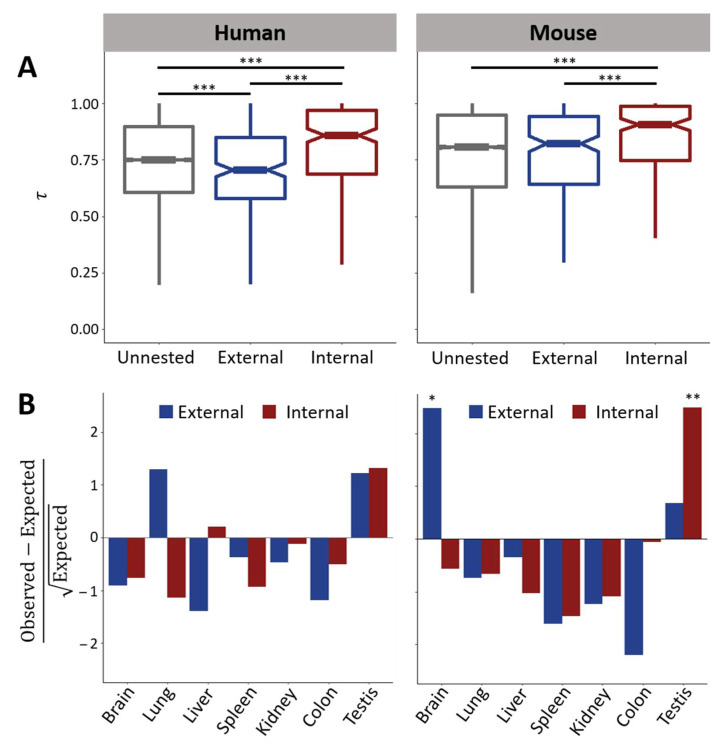
Expression breadths of external and internal protein-coding genes. (**A**) Distributions of tissue specificities (τ) across seven tissues in human (left) and mouse (right) unnested, external, and internal genes. Higher τ corresponds to greater tissue specificity. (**B**) Hanging chi-grams comparing observed numbers of human (**left**) and mouse (**right**) primary tissues of tissue-specific external and internal genes to expectations based on those of unnested genes. Positive and negative values indicate over-representations and under-representations, respectively. * *p* < 0.05, ** *p* < 0.01, and *** *p* < 0.001 (after Bonferroni corrections for Figure 1B; see Materials and Methods for details).

Previous studies have shown that young duplicate genes in mammals and many other animals and plants tend to be expressed primarily in male reproductive tissues [33,34,35,36,37,38,39]. However, if transcriptional interference drives the evolution of nested genes, then one would expect external and internal genes to be expressed in different tissues. Consistent with this hypothesis, studies of *Drosophila* nested genes have shown that whereas internal genes are often testis specific, external genes tend to be broadly expressed across several tissues [5,6]. To determine whether this is also true in mammals, I first examined distributions of the tissue specificity index τ [21] across seven tissues in human and mouse unnested, external, and internal genes (Figure 1A; see Materials and Methods for details). In both mammals, internal genes tend to be more tissue specific than either external or unnested genes, mirroring results in *Drosophila* [5,6]. Also consistent with *Drosophila* [5,6], human external genes are significantly more broadly expressed than unnested genes. However, this is not the case for mouse external genes, which have similar expression breadths as unnested genes. Nevertheless, both mammals display elevated tissue specificities of their internal nested genes, consistent with observations of young duplicate genes [33,34,35,36,37,38,39], as well as clear differences between expression breadths of external and internal genes, supporting the expectation under transcriptional interference. 

To further investigate expression breadths of nested protein-coding genes, I extracted all tissue-specific (τ > 0.9) [21] genes. Application of this cutoff yielded 40 external and 81 internal tissue-specific genes in human, and 69 external and 101 internal tissue-specific genes in mouse (Table 2). Of this subset, there are 18 cases for which both external and internal genes in a pair are tissue specific in human (Table S1), and 78 such cases in mouse (Table S2). Thus, there are similar over-representations of internal tissue-specific genes in both mammals, consistent with previous findings in *Drosophila* [5,6]. For each tissue, I compared observed numbers of tissue-specific nested genes to expectations based on unnested genes (Figure 1B; see Materials and Methods for details). In human, no statistically significant trends were uncovered, perhaps due to a lack of power from small sample sizes (Table 2), though there may be a preference for testis specificity among internal genes (*p* = 0.06). In mouse, there are larger and statistically significant over-representations of testis-specific internal and brain-specific external genes. Thus, though external genes tend to be more tissue specific in mouse than in human, the primary tissue in which mouse external genes are expressed (brain) differs from the primary tissue in which their internal genes are expressed (testis). Therefore, the results in both mammals suggest that external and internal genes are typically expressed in different tissues, as one might expect under transcriptional interference. 

### 3.3. Expression Divergence between Nested Protein-Coding Genes in Mammals

**Figure 2:**
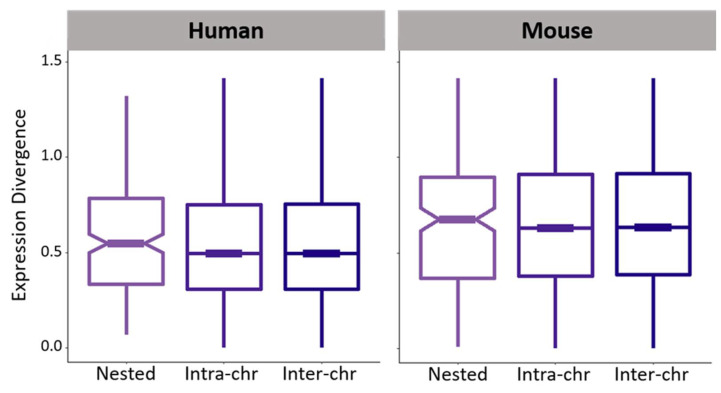
Expression divergence between nested, intra-chromosomal, and inter-chromosomal protein-coding genes. Distributions of Euclidian distances across seven tissues between gene pairs in human (left) and mouse (right). None of the pairwise differences between distributions are statistically significant (see Materials and Methods for details).

Last, I compared expression divergence across the seven tissues between pairs of nested, intra-chromosomal, and inter-chromosomal protein-coding genes (Figure 2; see Materials and Methods for details). In both mammals, expression divergence between nested genes is slightly elevated, but not significantly different from that between intra-chromosomal or inter-chromosomal genes. This result starkly contrasts the much higher expression divergence observed between *Drosophila* nested genes [6]. Perhaps not surprisingly, there is also no support in mammals for the rapid increase in expression divergence after nesting that was observed in *Drosophila* [6]. In particular, though numbers of nesting events are small in both mammals (Tables S3 and S4), expression divergence between these derived nested genes and their ancestral unnested orthologs are similar to one another, as well as to that between nested genes conserved in both mammals (Table S5; *p* > 0.05 for both comparisons, permutation tests; see Materials and Methods for details). Hence, expression divergence does not appear to substantially increase either immediately or long after gene nesting has occurred in mammals. This lack of expression divergence is consistent with lower selection efficiencies in mammals than in *Drosophila* [40]. Further, perhaps the relative deficiency of nested genes in mammals is reflective of a preference for eradicating new nesting events in their avoidance of transcriptional interference, as their abilities to diverge and accommodate new nested gene structures are more limited than those of *Drosophila*. 

## Figures and Tables

**Table 1:** Numbers of Simple Nested Protein-Coding Genes on the Same and Opposite Strands.

	Same	Opposite	Same vs. Opposite *
**Human**	76	220	$p=2.13\times 10^{-17}$
**Mouse**	55	193	$p=3.67\times 10^{-19}$
Human vs. Mouse **	p=0.37		

* Binomial tests (see Materials and Methods for details). ** Fisher’s exact test (see Materials and Methods for details).

**Table 2:** Numbers of Tissue-Specific External and Internal Protein-Coding Genes.

	External	Internal	External vs. Internal *
**Human**	40	81	$p=2.13\times 10^{-17}$
**Mouse**	69	101	$p=3.67\times 10^{-19}$
Human vs. Mouse **	p=0.22		

* Binomial tests (see Materials and Methods for details). ** Fisher’s exact test (see Materials and Methods for details).

## Data Availability

The data produced in this study are provided as Supplementary Materials (Tables S1–S5).

## Supplementary Materials

The following are available online at https://www.mdpi.com/article/10.3390/genes12091381/s1, Table S1: Nested protein-coding genes in human, Table S2: Nested protein-coding genes in mouse, Table S3: Protein-coding gene nesting events that occurred before the divergence of human and mouse lineages, Table S4: Protein-coding gene nesting events that occurred in the human lineage, Table S5: Protein-coding gene nesting events that occurred in the mouse lineage.
